# Letter from Mississippi

**Published:** 1871-09

**Authors:** 


CANTON, MISS., SEPTEMBER——, 1871.

DRS. POWELL & GOLDSMITH, ATLANTA, GA.:

*Messrs. Editors*—I appreciate the high position upon ethics, you have taken, hoping thereby, to elevate the Profession, which, unfortunately, I regret to say, does not occupy that high position in this country, that it is justly entitled. I trust you will pardon me for urging that you continue to keep before the Medical Profession that high stand-point which we may arrive at by a united effort. Also, I hope you will urge, through your columns, a higher degree of proficiency for graduation in our medical schools. Doctors are made too easily in this country, a longer term of pupilage should be insisted upon. To elevate the Profession, a more thorough system must be adopted. We *must look to our journals* to bring about this ehange. The enlightened age in which we live demands it, and the physical well-fare of the community calls loudly for it.

We take great pleasure in giving the above extract. It comes from a distant State—from Dr. A. H. Cage, Canton, Mississippi. We are pleased to know that THE COMPANIGN is meeting the approval of the Profession. Such letters we are receiving from every quarter. They show the animus of the Profession—the beating of the hearts of its true friends and admirers. The necessity of maintaining the dignity of our calling is forcing itself upon the attention of thinking physicians. Evils surround it on every side. A friend from another State, writes that the best talent of that State is leaving the Profession in despair. This will be the case everywhere unless a stand is made to rescue the Profession from its deplorable condition. We need union, determination and adhesion to principle. We must exalt the Profession. The ethics must be enforced and obeyed. The standard of medical education must be raised. The Profession must demand these things of its friends. Let the lines be drawn—the sooner the better. We urge upon the friends of professional honor everywhere, to labor day and night in the good cause, that science may be advanced, and the honor of medicine be preserved as a rich legacy to our children.

